# Thrombotic and bleeding complications in patients with chronic lymphocytic leukemia and severe COVID-19: a study of ERIC, the European Research Initiative on CLL

**DOI:** 10.1186/s13045-022-01333-0

**Published:** 2022-08-26

**Authors:** Darko Antic, Natasa Milic, Thomas Chatzikonstantinou, Lydia Scarfò, Vladimir Otasevic, Nina Rajovic, David Allsup, Alejandro Alonso Cabrero, Martin Andres, Monica Baile Gonzales, Antonella Capasso, Rosa Collado, Raul Cordoba, Carolina Cuéllar-García, Juan Gonzalo Correa, Lorenzo De Paoli, Maria Rosaria De Paolis, Giovanni Del Poeta, Maria Dimou, Michael Doubek, Maria Efstathopoulou, Shaimaa El-Ashwah, Alicia Enrico, Blanca Espinet, Lucia Farina, Angela Ferrari, Myriam Foglietta, Alberto Lopez-Garcia, José A. García-Marco, Rocío García-Serra, Massimo Gentile, Eva Gimeno, Maria Gomes da Silva, Odit Gutwein, Yervand K. Hakobyan, Yair Herishanu, José Ángel Hernández-Rivas, Tobias Herold, Gilad Itchaki, Ozren Jaksic, Ann Janssens, Olga B. Kalashnikova, Elżbieta Kalicińska, Arnon P. Kater, Sabina Kersting, Maya Koren-Michowitz, Jorge Labrador, Deepesh Lad, Luca Laurenti, Alberto Fresa, Mark-David Levin, Carlota Mayor Bastida, Lara Malerba, Roberto Marasca, Monia Marchetti, Juan Marquet, Biljana Mihaljevic, Ivana Milosevic, Fatima Mirás, Marta Morawska, Marina Motta, Talha Munir, Roberta Murru, Raquel Nunes, Jacopo Olivieri, Miguel Arturo Pavlovsky, Inga Piskunova, Viola Maria Popov, Francesca Maria Quaglia, Giulia Quaresmini, Gianluigi Reda, Gian Matteo Rigolin, Amit Shrestha, Martin Šimkovič, Svetlana Smirnova, Martin Špaček, Paolo Sportoletti, Oana Stanca, Niki Stavroyianni, Doreen Te Raa, Kristina Tomic, Sanne Tonino, Livio Trentin, Ellen Van Der Spek, Michel van Gelder, Marzia Varettoni, Andrea Visentin, Candida Vitale, Vojin Vukovic, Ewa Wasik-Szczepanek, Tomasz Wróbel, Lucrecia Yáñez San Segundo, Mohamed Yassin, Marta Coscia, Alessandro Rambaldi, Emili Montserrat, Robin Foà, Antonio Cuneo, Marc Carrier, Paolo Ghia, Kostas Stamatopoulos

**Affiliations:** 1 Lymphoma Center, Clinic for Hematology, University Clinical Center of Serbia, Belgrade, Serbia; 2 Faculty of Medicine, University of Belgrade, Belgrade, Serbia; 3 Department of Medical Statistics and Informatics, Faculty of Medicine, University of Belgrade, Belgrade, Serbia; 4 Hematology Department and HCT Unit, G. Papanicolaou Hospital, Thessaloniki, Greece; 5 Institute of Applied Biosciences, Centre for Research and Technology Hellas, Thessaloniki, Greece; 6 Università Vita-Salute San Raffaele and IRCC Ospedale San Raffaele, Milan, Italy; 7 Centre for Atherothrombosis and Metabolic Disease, Hull York Medical School, Hull, UK; 8 Haematology Department, Hospital Universitario de La Princesa, Madrid, Spain; 9 Department of Hematology and Central Hematology Laboratory, Inselspital, Bern University Hospital, University of Bern, Bern, Switzerland; 10 Hematology Department, University Hospital of Salamanca-IBSAL, Salamanca, Spain; 11 IRCSS Ospedale San Raffaele, Milan, Italy; 12 Department of Hematology, Hospital General Universitario, Valencia, Spain; 13 Fundación de Investigación del Hospital General Universitario, Valencia, Spain; 14 Department of Hematology, Health Research Institute IIS-FJD, Fundacion Jimenez Diaz University Hospital, Madrid, Spain; 15 Hematology Unit Terrassa Hospital, Terrassa, Spain; 16 Hospital Clínic de Barcelona, Barcelona, Spain; 17 Division of Internal Medicine, Hematology Unit, ASL Vercelli, Vercelli, Italy; 18 UOC Ematologia PO Vito Fazzi Lecce, Lecce, Italy; 19 Department of Biomedicine and Prevention Hematology, University Tor Vergata, Rome, Italy; 20 1st Internal Medicine Department, Propaedeutic, Hematology Clinical Trial Unit, National and Kapodistrian University of Athens, Athens, Greece; 21 Department of Internal Medicine – Hematology and Oncology, University Hospital, Brno, Czechia; 22 Department of Medical Genetics and Genomics, Faculty of Medicine, Masaryk University, Brno, Czechia; 23 Department of Haematology Athens Medical Center-Psychikon Branch, Athens, Greece; 24 Clinical Hematology Unit, Oncology Center, Faculty of Medicine, Mansoura University, Mansoura, 35516 Egypt; 25 Hospital Italiano La Plata, La Plata, Argentina; 26 Department of Hematology, Hospital del Mar, Barcelona, Spain; 27 Hematology, Fondazione IRCCS Istituto Nazionale Tumori, Milan, Italy; 28 Hematology Unit, Azienda Unità Sanitaria Locale – IRCCS, Reggio Emilia, Italy; 29 SC Ematologia, AO S. Croce e Carle, Cuneo, Italy; 30 Hematology Department, Hospital Universitario Puerta de Hierro-Majadahonda, Madrid, Spain; 31 Hematology Unit AO Cosenza, Cosenza, Italy; 32 Hematology Department, Portuguese Institute of Oncology Lisbon, Lisbon, Portugal; 33 Department of Hematology, Shamir Medical Center, Zerifin, Israel; 34 Sackler Faculty of Medicine, Tel Aviv University, Tel Aviv, Israel; 35 Hematology Center after Prof. Yeolyan MH RA, Yerevan, Armenia; 36 Department of Hematology, Tel Aviv Sourasky Medical Center and Sackler School of Medicine, Tel Aviv University, Tel Aviv, Israel; 37 Department of Hematology, Infanta Leonor University Hospital, Madrid, Spain; 38 Laboratory for Leukemia Diagnostics, Department of Medicine III, University Hospital, LMU Munich, Munich, Germany; 39 Division of Hematology, Rabin Medical Center, Petah Tikva, and the Sackler School of Medicine, Tel-Aviv University, Tel Aviv, Israel; 40 Department of Hematology, University Hospital Dubrava, Zagreb, Croatia; 41 Department of Hematology, Universitaire Ziekenhuizen Leuven, Leuven, Belgium; 42 Federal State Budgetary Educational Institution of Higher Education Academician I.P. Pavlov, First St. Petersburg State Medical University of the Ministry of Healthcare of Russian Federation, St. Petersburg, Russia; 43 Department and Clinic of Hematology, Blood Neoplasms and Bone Marrow Transplantation, Wroclaw Medical University, Pasteura Street 4, 50-367 Wrocław, Poland; 44 Department of Hematology, Cancer Center Amsterdam, Amsterdam University Medical Centers, University of Amsterdam, Amsterdam, The Netherlands; 45 Department of Hematology, Haga Teaching Hospital, The Hague, The Netherlands; 46 Hematology Department, Unit Research, Complejo Asistencial Universitario de Burgos, Burgos, Spain; 47 Department of Internal Medicine, Postgraduate Institute of Medical Education and Research, Chandigarh, India; 48 Sezione di Ematologia, Dipartimento di Scienze Radiologiche ed Ematologiche, Università Cattolica del Sacro Cuore, Rome, Italy; 49 Dipartimento di Diagnostica per Immagini, Radioterapia Oncologica ed Ematologia, Fondazione Policlinico Universitario Agostino Gemelli IRCCS, Rome, Italy; 50 Department of Internal Medicine, Albert Schweitzer Hospital, Dordrecht, The Netherlands; 51 Spanish Society of Haematology and Hemotherapy (SEHH: Sociedad Española de Hematología y Hemoterapia), Madrid, Spain; 52 Hematology and Stem Cell Transplant Center Marche Nord Hospital, Pesaro, Italy; 53 Section of Hematology, Department of Medical Sciences, University of Modena and Reggio E., Modena, Italy; 54 Hematology Unit & TMO Center, AO SS Antonio e Biagio e Cesare Arrigo, Alessandria, Italy; 55 Hematology Department, Ramón y Cajal University Hospital, Madrid, Spain; 56 Clinical Centre of Vojvodina, Faculty of Medicine, University of Novi Sad, Novi Sad, Serbia; 57 Hematology Department, Hospital Universitario 12de Octubre, Madrid, Spain; 58 Experimental Hematooncology Department, Medical University of Lublin, Lublin, Poland; 59 Hematology Department, St. John’s Cancer Center, Lublin, Poland; 60 S.C. Ematologia ASST Spedali Civili Brescia, Brescia, Italy; 61 Consultant Haematologist, St James’s Hospital, Leeds, LS9 7TF UK; 62 Hematology and Stem Cell Transplantation Unit, Ospedale Oncologico A. Businco, ARNAS “G. Brotzu”, Cagliari, Italy; 63 Hematology Clinic, ASUFC, Udine, Italy; 64 FUNDALEU, Clinical Research Center Buenos Aires, Buenos Aires, Argentina; 65 Consultative Hematology Department with a Day Hospital for Intensive High-Dose Chemotherapy, National Research Center for Hematology, Moscow, Russia; 66 Hematology Department, Colentina Clinical Hospital, Bucharest, Romania; 67 Department of Medicine, Section of Hematology, University of Verona, Verona, Italy; 68 Hematology, ASST Papa Giovanni XXIII, Bergamo, Italy; 69 Hematology Unit, Foundation IRCCS Ca’ Granda Ospedale Maggiore Policlinico of Milan, Milan, Italy; 70 St. Anna University Hospital, Ferrara, Italy; 71 Hematology Unit, Nepal Cancer Hospital and Research Center, Lalitpur, Nepal; 72 4th Department of Internal Medicine – Haematology, Faculty of Medicine in Hradec Králové, University Hospital and Charles University in Prague, Hradec Kralove, Czech Republic; 73 1st Department of Medicine - Hematology, First Faculty of Medicine, Charles University and General Hospital in Prague, Prague, Czech Republic; 74 Department of Medicine and Surgery, Institute of Hematology and Center for Hemato-Oncological Research, Ospedale S. Maria della Misericordia, Perugia, Italy; 75 Hematology Department from Coltea Clinical Hospital, “Carol Davila” University of Medicine and Pharmacy, Bucharest, Romania; 76 Department of Hematology, Gelderse Vallei Ede, Ede, The Netherlands; 77 Department of Hematology, Lymmcare, Cancer Center Amsterdam, Amsterdam University Medical Centers, University of Amsterdam, Amsterdam, The Netherlands; 78 Hematology and Clinical Immunology Unit, Department of Medicine, University of Padova, Padua, Italy; 79 Department of Internal Medicine, Rijnstate Hospital, Arnhem, The Netherlands; 80 Department Internal Medicine, Maastricht University Medical Center, Maastricht, The Netherlands; 81 Division of Hematology, Fondazione IRCCS Policlinico San Matteo, Pavia, Italy; 82 Division of Hematology, A.O.U. Cittàdella Salute e della Scienza di Torino and Department of Molecular Biotechnology and Health Sciences, University of Turin, Turin, Italy; 83 Department Hematooncology and Bone Marrow Transplantation, Medical University in Lublin, Lublin, Poland; 84 University Hospital and Research Institute of Marqués de Valdecilla (IDIVAL), Santander, Spain; 85 Hematology Section, Department of Medical Oncology, National Center for Cancer Care and Research, Doha, Qatar; 86 Hematology, Department of Translational and Precision Medicine, Sapienza University, Rome, Italy; 87 Department of Medicine, Ottawa Hospital Research Institute, University of Ottawa, Ottawa, ON K1H 8L6 Canada

**Keywords:** CLL, COVID-19, Thrombosis, Bleeding, D-dimer, Anticoagulation therapy, Thromboprophylaxis, LMWH, Age

## Abstract

**Background:**

Patients with chronic lymphocytic leukemia (CLL) may be more susceptible to COVID-19 related poor outcomes, including thrombosis and death, due to the advanced age, the presence of comorbidities, and the disease and treatment-related immune deficiency. The aim of this study was to assess the risk of thrombosis and bleeding in patients with CLL affected by severe COVID-19.

**Methods:**

This is a retrospective multicenter study conducted by ERIC, the European Research Initiative on CLL, including patients from 79 centers across 22 countries. Data collection was conducted between April and May 2021. The COVID-19 diagnosis was confirmed by the real-time polymerase chain reaction (RT-PCR) assay for SARS-CoV-2 on nasal or pharyngeal swabs. Severe cases of COVID-19 were defined by hospitalization and the need of oxygen or admission into ICU. Development and type of thrombotic events, presence and severity of bleeding complications were reported during treatment for COVID-19. Bleeding events were classified using ISTH definition. STROBE recommendations were used in order to enhance reporting.

**Results:**

A total of 793 patients from 79 centers were included in the study with 593 being hospitalized (74.8%). Among these, 511 were defined as having severe COVID: 162 were admitted to the ICU while 349 received oxygen supplementation outside the ICU. Most patients (90.5%) were receiving thromboprophylaxis. During COVID-19 treatment, 11.1% developed a thromboembolic event, while 5.0% experienced bleeding. Thrombosis developed in 21.6% of patients who were not receiving thromboprophylaxis, in contrast to 10.6% of patients who were on thromboprophylaxis. Bleeding episodes were more frequent in patients receiving intermediate/therapeutic versus prophylactic doses of low-molecular-weight heparin (LWMH) (8.1% vs. 3.8%, respectively) and in elderly. In multivariate analysis, peak D-dimer level and C-reactive protein to albumin ratio were poor prognostic factors for thrombosis occurrence (OR = 1.022, 95%CI 1.007‒1.038 and OR = 1.025, 95%CI 1.001‒1.051, respectively), while thromboprophylaxis use was protective (OR = 0.199, 95%CI 0.061‒0.645). Age and LMWH intermediate/therapeutic dose administration were prognostic factors in multivariate model for bleeding (OR = 1.062, 95%CI 1.017–1.109 and OR = 2.438, 95%CI 1.023–5.813, respectively).

**Conclusions:**

Patients with CLL affected by severe COVID-19 are at a high risk of thrombosis if thromboprophylaxis is not used, but also at increased risk of bleeding under the LMWH intermediate/therapeutic dose administration.

## Background

High rates of venous thromboembolism (VTE), predominantly pulmonary embolism (PE), have been documented in patients with coronavirus disease 2019 (COVID-19), particularly in critically ill patients admitted to the intensive care unit (ICU) [1, 2]. Despite the use of prophylactic or even therapeutic doses of anticoagulation therapy, thromboembolic complications have developed in many patients, implying that the risk of thrombotic complications remains high despite treatment, while also prompting the use of higher than usual doses of anticoagulants in hospital settings [3, 4]. The pathophysiology of this prothrombotic state is multifactorial and not yet completely elucidated. However, immune dysregulation [5], endotheliopathy [6] and coagulopathy [7] are distinctive elements of COVID-19 that have a major impact on thrombosis development.

The use of anticoagulation therapy, particularly at intermediate and therapeutic doses, is associated with an increased risk of haemorrhagic events [8]. Initial reports revealed limited evidence of COVID-19 therapy-related bleeding, but more data concerning the risk of bleeding are accumulating, particularly as regards the use of therapeutic doses of anticoagulation therapy [9]. Considering the ongoing pandemic and its impact on vulnerable groups of patients, it is of immense importance to assess the actual rate of both thrombotic and bleeding events in specific patient populations.

Chronic lymphocytic leukemia (CLL) is the most prevalent leukemia in the western world [10]. Patients with CLL may be more susceptible to COVID-19-related poor outcomes, such as thrombosis and death [11]. Due to advanced age, the presence of various comorbidities, and the inherent immune deficiency of patients with CLL, there is a need for a robust analysis of the effects of patient and CLL-related characteristics, and thromboprophylactic therapy to define the optimal management of these patients during the COVID-19 pandemic.

In this retrospective international multicenter study, we assessed the risk of thrombosis as well as the risk of bleeding due to the administration of thromboprophylaxis in severely ill patients with CLL and COVID-19 and sought to identify potential predictors of thrombosis.

## Methods

### Data collection

This is a retrospective multicenter study conducted by ERIC, the European Research Initiative on CLL, including patients from 79 centers across 22 countries. Data collection was conducted between April and May 2021. The study was approved by the ethics committees of the collaborating institutions. This cohort of CLL patients represents a subgroup of recently published ERIC and Campus CLL study [12].

In adherence to the international standard of practice, the criteria for COVID-19 diagnosis were positive real-time polymerase chain reaction (RT-PCR) assay for SARS-CoV-2 on nasal or pharyngeal swabs. Patients whose radiological or clinical assessments were suspicious of COVID-19, but had a negative swab test, were not included in the study.

The CLL diagnostic procedures, patient assessment, clinical decisions, and actual treatment were performed by local hematology teams following international CLL guidelines [13, 14].

The following patient clinical characteristics and laboratory data were obtained in the survey: baseline demographics; CLL diagnosis date; treatment status; presence, number, and type of comorbidities [cumulative illness rating scale (CIRS)], date of COVID-19 diagnosis; symptoms, treatment, and outcome of COVID-19; need for and duration of hospitalization; type of ward (intensive care unit (ICU) vs. non-ICU ward); peak absolute lymphocyte count (ALC); peak C-reactive protein (CRP); nadir albumin level; peak D-dimer level; use, type, and dosage of thromboprophylaxis; development and type of thrombotic events, presence and severity of bleeding complications during the hospitalization for COVID-19. Dosage of low-molecular weight heparin (LMWH) was defined as: prophylactic dose 50 IU/kg s.c. daily, intermediate dose 100 IU/kg s.c. daily and therapeutic dose 200 IU/kg s.c. daily. The use of extended thromboprophylaxis after discharge from hospitalization was defined as prophylactic dosage of anticoagulation administered to patients at high risk for VTE for up to 39 days post-discharge [15]. Thrombotic events were classified as: pulmonary embolism (PE), deep vein thrombosis (DVT), stroke, myocardial infarction (MI), line associated thrombosis, extracorporeal circuit clotting in haemodialysis or ECMO lines and pernio-like skin lesions. Bleeding events have been classified as major using the International Society on Thrombosis and Haemostasis (ISTH) definition, whereas all non-major bleeding events were classified as minor [16].

To eliminate collection bias, we restricted our analysis to the group of patients who were considered to have severe COVID-19. Severe COVID-19 was defined as hospitalization and need of oxygen or admission into ICU while nonsevere/mild COVID-19 was defined as confinement at home or hospitalization without need of oxygen [12].

In order to enhance reporting, we used the Strengthening the Reporting of Observational studies in Epidemiology (STROBE) checklist, which is an evidence-based, minimum set of recommendations for reporting observational studies in biomedical sciences [17].

### Statistical analysis

Numerical data were presented as means with standard deviation or with median with 25‒75th percentile. Categorical variables are summarized as absolute numbers with percentages or rates with corresponding 95% confidence intervals (CIs). The Kolmogorov–Smirnov test was used to assess the normality of data distribution. Student’s *t*-test for independent samples or the Mann‒Whitney *U* test was applied for numerical variables according to the data distribution. For categorical variables, Pearson’s chi square analysis and Fisher’s exact test were used. Predictors of thrombosis and bleeding occurrence during treatment were identified using univariate and multivariate logistic regression analyses, and presented with odds ratios (ORs) and corresponding 95% CIs. Variables were selected based on their associations with increased risk for thrombosis and bleeding (*p* < 0.10; univariate analysis) or known relevance, and were included in the variable pool for a stepwise-regression model. No imputation methods were used in analysis. If an outcome was missing, the patient data was excluded from the analysis. Receiver operating characteristic (ROC) curve analysis was used to test the model’s discrimination performance based on sensitivity and specificity. Statistical analysis was performed using IBM SPSS statistical software (SPSS for Windows, release 25.0, SPSS, Chicago, IL, USA).

## Results

**Fig. 1:**
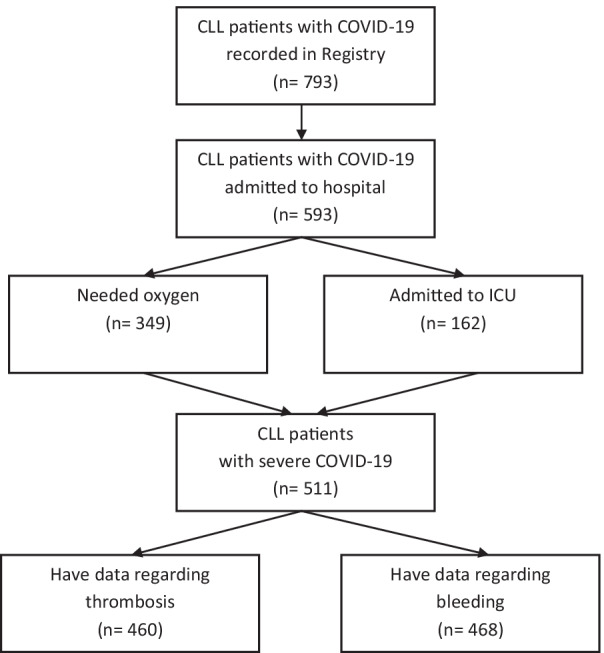
Flow chart of study participants

We collected data from a total of 793 patients with SARS-CoV-2 infection (Fig. 1). Most patients (742; 93.6%) were diagnosed with CLL, while 36 (4.5%) and 15 (1.9%) were diagnosed with small lymphocytic lymphoma (SLL) and monoclonal B-cell lymphocytosis (MBL), respectively. The patients were predominantly men (69.5%), with a median age of 69 years (25th‒75th percentile: 61‒77 years). Five hundred and ninety-three (74.8%) patients were admitted to the hospital. Among these, 349 needed oxygen supplementation outside the ICU, while 162 were admitted to the ICU. Further analysis was restricted to this group of patients (*n* = 511) who were considered to have severe COVID-19. Median follow-up time i.e., duration of hospitalization for CLL patients with severe COVID-19 was 16 days (25–75th percentile, 10–26 days).

CLL patients with severe COVID-19 were predominantly male (69.5%), with a median age of 70 years (25th‒75th percentile, 63‒79 years). Most cases had a significant burden of two or more comorbidities (62.9%), with hypertension (49.9%), diabetes (22.2%), coronary artery disease (12.2%), arrhythmias (9.8%), and other cardiovascular comorbidities and non-hematological malignancy (8.8% and 7.5%, respectively) being the most common. The reported median CIRS score was 4 (25th‒75th percentile, 2‒7). Forty-five percent were treatment naive (“watch and wait”), while 55% had received at least one line of CLL therapy (median, 1; range 1‒5). At the time of COVID-19 diagnosis, 34.3% of patients were receiving active CLL therapy, most commonly Bruton tyrosine kinase inhibitors (BTKi’s) (54.9%).

Out of 511 CLL patients with severe COVID-19, data regarding thromboembolic events were available for 460, while data regarding bleeding were available for 468 patients. In this cohort of severe COVID-19 patients with CLL, 11.1% of patients (51/460, 95%CI 8–14%) developed thromboembolic events during treatment for COVID-19: 37 patients developed PE (8.0%), 7 patients deep vein thrombosis (1.5%), 5 patients stroke (1.1%), 2 myocardial infarction (0.4%), one patient developed line associated thrombosis and one developed pernio-like skin lesions. There were no extracorporeal circuit clotting in haemodialysis or ECMO lines. A total of 4.1% (19/460) of deaths were suspected to be related to thrombosis (Table 1). Twenty-three patients (23/468, 4.9%, 95%CI 3–7%) experienced bleeding during COVID-19 treatment (12 major bleeding; 11 non-major bleeding cases). Detailed information about patient characteristics according to thrombosis and bleeding status is presented in Table 2. There were no differences in baseline patient characteristics between patients who developed thrombosis during COVID-19 treatment versus those who did not develop thrombosis, with the exception of the presence of other cardiovascular diseases. Patients who experienced bleeding were significantly older than patients who did not experience bleeding.

**Table 1:** Thrombosis and bleeding in CLL patients during hospitalization for severe COVID-19

	*n*/*N*	95% CI
Thrombosis overall*	51/460 (0.11)	0.08–0.14
Pulmonary embolism	37/51	
Deep vein thrombosis	7/51	
Ischaemic stroke	5/51	
Myocardial infarction	2/51	
Line associated thrombosis	1/51	
Pernio-like skin lesions	1/51	
Thrombosis-related death	19/460 (0.04)	0.02–0.06
Bleeding overall	23/468 (0.05)	0.03–0.07
Major	12/23	
Gastrointestinal	6/12	
CNS/haemorrhagic stroke	3/12	
Intramuscular	3/12	
Minor*	11/23	
Epistaxis	5/11	
Skin	4/11	
Genitourinary	2/11	
Gastrointestinal	1/11	
Conjuctival	1/11	

*Two patients had more than one event

**Table 2:** Characteristics of the present cohort according to thrombosis and bleeding status

	Thrombosis		Bleeding	
	No (*n* = 409)	Yes (*n* = 51)	No (*n* = 445)	Yes (*n* = 23)
Gender, male, *n*%	283/409 (69.2)	35/51 (68.6)	313/445 (70.3)	15/23 (65.2)
Age, median (25–75th percentile)	70 (63‒79)	67 (61‒77)	69 (63–78)	78 (66–86)*
Smoking				
Never, *n*%	253/378 (66.9)	31/48 (64.6)	275/414 (66.4)	13/21 (61.9)
Ex-smoker, *n*%	96/378 (25.4)	13/48 (27.1)	108/414 (26.1)	6/21 (28.6)
Current smoker, *n*%	29/378 (7.7)	4/48 (8.3)	31/414 (7.5)	2/21 (9.5)
Obesity, *n*%	71/390 (18.2)	8/50 (16.0)	73/425 (17.2)	5/21 (23.8)
Presence of any comorbidity, *n*%	339/408 (83.1)	45/50 (90.0)	367/443 (82.8)	19/23 (82.6)
Number of comorbidities				
No comorbidities, *n*%	69/408 (16.9)	5/50 (10.0)	76/443 (17.2)	4/23 (17.4)
1 comorbidity, *n*%	86/408 (21.1)	14/50 (28.0)	91/443 (20.5)	6/23 (26.1)
> 2 comorbidities, *n*%	253/408 (62.0)	31/50 (62.0)	276/443 (62.3)	13/23 (56.5)
Type of comorbidities				
Other respiratory, *n*%	25 (6.1)	6 (12.0)	33 (7.4)	2 (8.7)
Asthma, *n*%	12 (2.9)	1 (2.0)	14 (3.2)	0 (0)
COPD, *n*%	26 (6.4)	1 (2.0)	30 (6.8)	1 (4.3)
Other cardiovascular, *n*%	31 (7.6)	8 (16.0)*	39 (8.8)	0 (0)
Cardiac failure, *n*%	12 (2.9)	1 (2.0)	11 (2.5)	2 (8.7)
Arrhythmias, *n*%	35 (8.6)	8 (16.0)	40 (9.0)	4 (17.4)
Coronary artery disease, *n*%	43 (10.5)	4 (8.0)	47 (10.6)	1 (4.3)
Hypertension, *n*%	202 (49.5)	23 (46.0)	216 (48.8)	12 (52.2)
Diabetes, *n*%	95 (23.3)	11 (22.0)	101 (22.8)	4 (17.4)
Other hematological malignancy, *n*%	6 (1.5)	2 (4.0)	6 (1.4)	1 (4.3)
Other non-hematological malignancy, *n*%	30 (7.4)	5 (10.0)	35 (7.9)	2 (8.7)
Chronic renal disease, *n*%	26 (6.4)	4 (8.0)	27 (6.1)	2 (8.7)
CIRS, median (25–75th percentile)	4 (2‒7)	4 (2‒7)	4 (2–7)	4 (2–7)

*COPD* chronic obstructive pulmonary disease, *CIRS* cumulative illness rating scale

*p < 0.05

Patients with CLL and severe COVID-19 presented with fever (82.6%), and respiratory symptoms, including dyspnea (60.6%) and cough (53.8%). Other symptoms included fatigue (22.1%), headache (5.7%), myalgias/arthralgias (9.5%), anosmia/ageusia (4.9%), and gastrointestinal symptoms (10.1%). Other symptoms were observed in 15.6% patients. Data regarding specific symptoms manifested during COVID-19 are presented according to thrombosis and bleeding status in Table 3. There was no statistically significant difference in symptoms between the groups.

**Table 3:** Presenting symptoms of severe COVID-19 according to thrombosis and bleeding status of CLL patients with COVID-19

	Thrombosis		Bleeding	
	No (*n* = 409)	Yes (*n* = 51)	No (*n* = 445)	Yes (*n* = 23)
Fever	340/408 (83.3)	41/51 (80.4)	368/444 (82.9)	19/23 (82.6)
Dyspnea	241/406 (59.4)	33/51 (64.7)	264/441 (59.9)	15/23 (65.2)
Cough	223/408 (54.7)	25/51 (49.0)	239/444 (53.8)	11/23 (47.8)
Fatigue	86/408 (21.1)	9/51 (17.6)	94/444 (21.2)	4/23 (17.4)
Headache	24/408 (5.9)	4/51 (7.8)	23/444 (5.2)	3/23 (13.0)
GI symptoms	46/408 (11.3)	3/51 (5.9)	45/444 (10.1)	4/23 (17.4)
Anosmia/Ageusia	20/408 (4.9)	4/51 (7.8)	21/444 (4.7)	2/23 (8.7)
Myalgias/Arthralgias	38/408 (9.3)	5/51 (9.8)	41/444 (9.2)	2/23 (8.7)

*GI* gastrointestinal

*p < 0.05

One hundred and seventy five (34.3%) patients were receiving active CLL-directed therapy while ill with COVID-19 though, 140 (80.5%) stopped the CLL treatment after the infection. BTK inhibitors (*n* = 95) were the most common therapy used (54.9% of patients receiving CLL therapy). Neither continuation nor discontinuation of BTKi in CLL patients with COVID-19 infection impacted thrombosis and bleeding occurrence in patients with CLL (Table 4). Venetoclax was administered as monotherapy in 21 patients, and in combination with anti-CD20 monoclonal antibodies in 12 patients. A minority of patients received other therapies, including anti-CD20 monoclonal antibody monotherapy (*n* = 5) and phosphatidylinositol-3-kinase (PI3K) inhibitors monotherapy (*n* = 5), while a combination of anti-CD20 monoclonal antibodies and PI3K inhibitors received one patient. Fifteen patients received either chemotherapy or chemoimmunotherapy. Corticosteroids for CLL were administered to 12.0%.

**Table 4:** CLL-directed therapy and COVID-19 management strategies according to thrombosis and bleeding status of CLL patients with COVID-19

	Thrombosis		Bleeding	
	No (*n* = 409)	Yes (*n* = 51)	No (*n* = 445)	Yes (*n* = 23)
On CLL treatment at the time of COVID-19	132/408 (32.4)	21/51 (41.2)	149/444 (32.7)	11/23 (47.8)
On treatment with corticosteroids for CLL or other disease	46/397 (11.6)	6/51 (11.8)	49/432 (11.3)	3/23 (13.0)
Anti-CD20 at the time of COVID-19	27/406 (6.7)	3/51 (5.9)	25/442 (5.7)	2/23 (8.7)
Type of CLL treatment at the time of COVID-19				
BTKi only	69/130 (53.1)	10/21 (47.6)	83/147 (56.5)	4/11 (36.4)
Venetoclax	14/130 (10.8)	4/21 (19.0)	16/147 (10.9)	4/11 (36.4)
Venetoclax + Anti-CD20	11/130 (8.5)	1/21 (4.8)	9/147 (6.1)	2/11 (18.2)
PI3K inhibitors	5/130 (3.8)	0/21 (0.0)	5/147 (3.4)	0/11 (0)
Anti-CD20 only	4/130 (3.1)	1/21 (4.8)	4/147 (2.7)	0/11 (0)
Chemotherapy	10/130 (7.7)	2/21 (9.5)	11/147 (7.5)	1/11 (9.1)
Chemoimmunotherapy	12/130 (9.2)	2/21 (9.5)	13/147 (8.8)	0/11 (0)
BTKi + Venetoclax	2/130 (1.5)	0/21 (0.0)	2/147 (1.4)	0/11 (0)
Steroids only	3/130 (2.3)	1/21 (4.8)	4/147 (2.7)	0/11 (0)
Managing CLL treatment				
Continued as planned	25/131 (19.1)	5/21 (23.8)	30/148 (20.3)	2/11 (18.2)
Replaced with another treatment	0/131 (0)	1/21 (4.8)	0/148 (0)	1/11 (9.1)
Stopped treatment	106/131 (80.9)	15//21 (71.4)	118/148 (79.7)	8/11 (72.7)
Managing BTKi treatment				
BTKi at the time of COVID-19	71/130 (54.6)	10/21 (47.6)	85/147 (57.8)	4/11 (36.4)
Continued BTKi as planned	20/71 (28.2)	3/10 (30.0)	24/85 (28.2)	1/4 (25.0)
Stopped BTKi treatment	51/71 (71.8)	7/10 (70.0)	61/85 (71.8)	3/4 (75.0)
Pharmacological treatment for COVID-19				
Convalescent hyperimmune plasma	28/304 (9.2)	5/37 (13.5)	30/328 (9.1)	5/18 (27.8)*
Antivirals	160/358 (44.7)	24/45 (53.3)	181/390 (46.4)	9/22 (40.9)
Hydroxychloroquine or similar	139/356 (39.0)	14/43 (32.6)	150/385 (39.0)	8/22 (36.4)
Azithromycin	143/351 (40.7)	17/43 (39.5)	158/380 (41.6)	7/22 (31.8)
Steroids	320/390 (82.1)	47/49 (95.9)*	354/423 (83.7)	21/23 (91.3)
Anti-IL6 or anti-IL6R	57/349 (16.3)	19/45 (42.2)*	70/380 (18.4)	7/22 (31.8)
ICU admission	109/408 (26.7)	27/51 (52.9)*	128/444 (28.8)	9/23 (39.1)
Supportive therapy, ECMO	2/409 (0.5)	2/51 (3.9)*	2/445 (0.4)	2/23 (8.7)*

*CLL* chronic lymphocytic leukemia, *BTKi* Bruton tyrosine kinase inhibitors, *COVID-19* coronavirus disease 2019, *PI3K* phosphatidylinositol-3-kinase inhibitors, *ICU* intensive care unit, *ECMO* Extracorporeal membrane oxygenation

*p < 0.05

Pharmacological treatment for COVID-19 included antivirals (45.6%), azithromycin (40.5%), hydroxychloroquine or similar drugs (37.9%), anti-IL6 or anti-IL6R monoclonal antibodies (19.1%), and convalescent hyperimmune plasma (10.1%). Steroids were administered to 83.4% of patients. Extracorporeal membrane oxygenation (ECMO) was used in 5 patients (1%). CLL-directed therapy and COVID-19 management strategies according to thrombosis and bleeding status are presented in Table 4. Steroids use for COVID-19, anti-IL6 or anti-IL6R treatment and admission to ICU were more common among patients who developed thrombosis in contrast to patients who did not develop thrombosis. Use of convalescent hyperimmune plasma was more common among patients who experienced bleeding in contrast to patients who did not experience bleeding. Use of supportive ECMO therapy was more common among patients who developed both thrombosis and bleeding.

The biochemical characteristics of the patients according to thrombosis and bleeding status are shown in Table 5. Peak D-dimer level was significantly higher in patients who developed thrombosis in contrast to patients who did not develop thrombosis, as well as in patients who experienced bleeding in contrast to patients who did not experience bleeding.

**Table 5:** Biochemical characteristics of the patients according to thrombosis and bleeding status

	Thrombosis		Bleeding	
	No	Yes	No	Yes
ALC (peak), × 10^9^/L	14.20	15.00	13.18	14.20
	(3.80‒52.00)	(1.90‒40.24)	(3.70–50.60)	(1.50–40.24)
Albumin (nadir), g/dL	3.20	3.10	3.20	3.05
	(2.80‒3.80)	(2.70‒3.60)	(2.80–3.80)	(2.82–3.50)
CRP, mg/L	21.76	25.00	22.75	23.40
(peak) (× times the ULN)	(11.40‒36.80)	(14.80‒41.73)	(11.80–37.20)	(9.91–35.48)
CAR	7.01	8.26	7.23	7.45
	(3.55‒11.83)	(5.15‒16.72)	(3.70–12.39)	(4.20–15.61)
D-dimer, mg/L	2.82	9.76	2.88	6.11
(peak) (× times the ULN)	(1.65‒6.53)	(3.36‒33.20)*	(1.64–7.44)	(3.12–31.76)*

Data are presented as median with 25–75th percentile; *p < 0.05

*ALC* absolute lymphocyte count, *IQR* interquartile range, *CRP* C-reactive protein, *CAR* C-reactive protein to albumin ratio, *ULN* upper limit of normal

Most patients (90.5%) were receiving thromboprophylaxis for COVID-19: 85.9% received LMWH, 3.6% received direct oral anticoagulants (DOACs), and 1.1% aspirin. Five patients treated with ECMO and three patients on haemodialysis were switched to unfractionated heparin (UFH) after initial LMWH approach. Thrombosis developed in 21.6% of patients who were not receiving thromboprophylaxis in contrast to 10.6% of patients who were on thromboprophylaxis (*p* = 0.043). Prophylactic dose was administered to 68.1%, intermediate to 14.3% and therapeutic to 17.7% of patients who received LMWH. Patients receiving intermediate/therapeutic doses of LMWH experienced more frequent thrombosis than patients who received prophylactic doses (22/126, 17.5% vs. 18/261, 6.9%, respectively) (*p* = 0.001), and experienced more frequent bleeding (10/124, 8.1% vs. 10/262, 3.8%, respectively) (*p* = 0.079). Extended thromboprophylaxis was administered to 26.8% of patients.

In univariate logistic regression analysis, admission to ICU, anti-IL6 or anti-IL6R treatment and steroids use for COVID-19 were predictive of thrombosis occurrence, (*p* < 0.001, OR = 3.086, 95%CI 1.707‒5.578; *p* < 0.001, OR = 3.744, 95%CI 1.942–7.215 and *p* = 0.026, OR = 5.141, 95%CI 1.220–21.665, respectively). High C-reactive protein to albumin ratio and D-dimer values were also predictive of thrombosis occurrence (*p* = 0.009, OR = 1.030, 95%CI 1.007‒1.052 and *p* = 0.002, OR = 1.016, 95%CI 1.006‒1.027, respectively). Thromboprophylaxis was protective factor for thrombosis occurrence (*p* = 0.049, OR = 0.428, 95%CI 0.184‒0.996). Presence of other cardiovascular diseases was of borderline significance (*p* = 0.050, OR = 2.316, 95%CI 1.000‒5.366). In multivariate analysis, peak D-dimer level, high C-reactive protein to albumin ratio and anti-IL6 or anti-IL6R treatment were poor prognostic factors for thrombosis occurrence (*p* = 0.005, OR = 1.022, 95%CI 1.007‒1.038; *p* = 0.042, OR = 1.025, 95%CI 1.001‒1.051 and *p* = 0.018, OR = 2.654, 95%CI 1.182‒5.958), in contrast to thromboprophylaxis use that was protective (*p* = 0.007, OR = 0.199, 95%CI 0.061‒0.645) (Table 6). In univariate logistic regression analysis, age (*p* = 0.012, OR = 1.055, 95%CI 1.012–1.100) and convalescent hyperimmune plasma (*p* = 0.017, OR = 3.821, 95%CI 1.275–11.450) were predictive of bleeding, while use and LMWH intermediate/therapeutic dose use was of borderline significance (*p* = 0.078, OR = 2.150, 95%CI 0.917–5.041). In multivariate analysis, age (*p* = 0.007, OR = 1.062, 95%CI 1.017–1.109) and LMWH intermediate/therapeutic dose (*p* = 0.044, OR = 2.438, 95%CI 1.023–5.813) were prognostic factors for bleeding. Continuation versus discontinuation of BTKi was not predictive of thrombosis or bleeding occurrence in the patients with CLL who were receiving BTKi at the time of severe COVID-19 infection (*p* < 0.05).

**Table 6:** Univariate and multivariate logistic regression analyses with thrombosis and bleeding as dependent variable

Variable	Univariate			Multivariate		
	OR	95% CI for OR	*p*	OR	95% CI for OR	*p*
*Thrombosis*						
Steroids for COVID-19	5.141	1.220–21.665	0.026			
Anti-IL6 or anti-IL6R	3.744	1.942–7.215	< 0.001	2.654	1.182–5.958	0.018
Admission to ICU	3.086	1.707–5.578	< 0.001			
D-dimer (×times the ULN)	1.016	1.006–1.027	0.002	1.022	1.007–1.038	0.005
CAR	1.030	1.007–1.052	0.009	1.025	1.001–1.051	0.042
Thromboprophylaxis	0.428	0.184–0.996	0.049	0.199	0.061–0.645	0.007
Other cardiovascular diseases	2.316	1.000–5.366	0.050			
Continued vs. stopped BTKi	1.157	0.433–3.092	0.772			
*Bleeding*						
Age	1.055	1.012–1.100	0.012	1.062	1.017–1.109	0.007
Convalescent hyperimmune plasma use	3.821	1.275–11.450	0.017			
LMWH intermediate/therapeutic dose use	2.150	0.917–5.041	0.078	2.438	1.023–5.813	0.044
Continued vs. stopped BTKi	1.086	0.342–3.452	0.888			

*IL-6* interleukin 6, *ULN* upper limit of normal, *CAR* C-reactive protein to albumin ratio, *LMWH* low molecular weight heparin, *BTKi* Bruton tyrosine kinase inhibitors

**Fig. 2:**
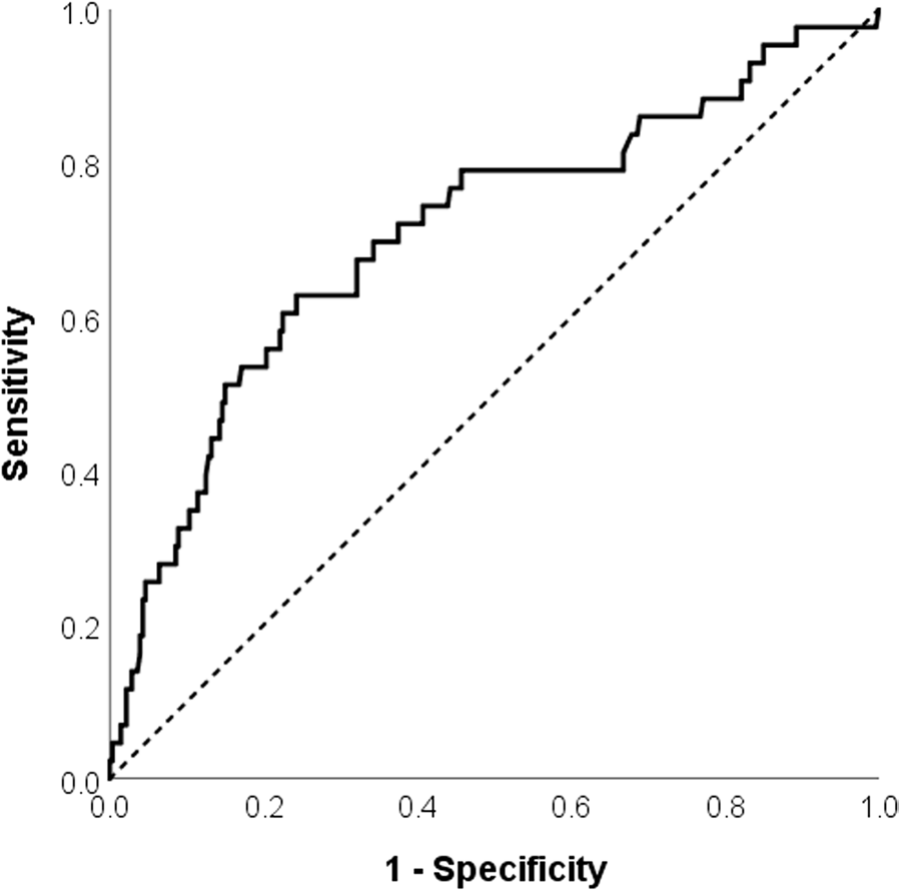
Receiver operating characteristic curve for D-dimer in distinguishing CLL patients with COVID-19, with and without thrombosis

Figure 2 presents the ROC curve for D-dimer in distinguishing CLL patients with COVID-19, with and without thrombosis (AUC = 0.709, *p* < 0.001). At a cut-off D-dimer value of 4 × ULN, the sensitivity and specificity were 72% and 63%, respectively.

## Discussion

CLL is the most prevalent leukemia in the western world, hence the need for improved understanding of COVID-19 in this group of patients is essential, particularly since patients with CLL are at higher risk of adverse outcomes of COVID-19 [11]. Against this background, data about the risk for TE events and bleeding complications in CLL patients with COVID-19 is scarce.

In the present cohort, the rate of thromboembolic events in CLL patients with severe COVID-19 was 11.1% (51/460), with PE being the most frequent (8.0%, 37/460). No significant differences were observed in CLL patients with or without thrombosis in terms of baseline patient characteristics, comorbidities and COVID-19-related symptoms. The published data by Chatzikonstantinou et al. [12] reported the VTE rate of 6.2% in the study that included 941 CLL patients with COVID-19. The study of 124 patients with various hematological malignancies [18], of whom 21 were patients with CLL, reported the rate of VTE of 8%, while the rate of composite thrombotic events (arterial and venous) was 13.4%. Besides the limitation of small patient numbers, direct comparison of this study and ours is of questionable relevance because the higher rate of cumulative thrombotic events in the former could be due to inclusion of particular hematological malignancies with well-established higher risk for thrombosis (e.g., plasma cell dyscrasia and myeloproliferative neoplasms). Comparisons with the general population with COVID-19 are also hindered by various confounding factors, not least of which is the fact that the rate of thrombosis depends largely on disease severity and, consequently, the hospital department (ICU vs. general ward): indeed, the disclosed rates of VTE in critically ill patients in ICU vary between 25 and 69%, in contrast to 7% in general wards [19]. In a systematic review and meta-analysis, the prevalence of VTE in non-ICU and ICU patients were 7.9% and 22.7%, respectively, while the prevalence of PE in non-ICU and ICU patients were 3.5% and 13.7%, respectively [20].

When diagnosed with COVID-19, 175 (34.3%) patients of the present cohort were receiving active CLL-directed therapy. BTK inhibitors were the most frequent CLL-directed therapies, followed by venetoclax. There were no significant differences regarding the type and (dis)continuation of CLL treatment between CLL patients with or without thromboembolic events related to COVID-19, including BTKi. Several possible reasons could account for this finding. First, no CLL-directed specific treatment was associated with an increased risk of thromboembolic events. Second, the treatment was stopped in the majority of CLL patients (80.5%) after the COVID-19 diagnosis was established. Third, the potential beneficial effect of BTK inhibitors on the amelioration of the COVID-19 clinical course [21] was principally due to the modulation of immunological response [22, 23], other than through the notable platelet inhibition effect [24] of BTK inhibitors. Lastly, a small number of CLL patients included in the study were treated with therapeutic options other than BTK inhibitors, which somehow limited the statistical analysis.

Focusing on the potential impact of pharmacological treatment for COVID-19 on the occurrence of thrombosis in the present cohort, patients who were administered corticosteroid therapy and anti-IL6 or anti-IL6R monoclonal antibody were significantly more often diagnosed with thromboembolism. Further, in univariate logistic regression analysis, admission to ICU and use of anti-IL6/anti-IL6R and corticosteroids were predictive of thrombosis occurrence. Anti-IL6 or anti-IL6R monoclonal antibodies have been extensively used in order to ameliorate the hyperinflammatory state. A previous report [25] pointed out a transient surge in D-dimer levels and an increased risk of death secondary to thromboembolism. The limitations of that study were the small number of patients (*n* = 24), the retrospective nature of the study, and the non-specified severity of COVID-19. Overall, further investigation is warranted regarding the possible relationship between the use of anti-IL6 or anti-IL6R monoclonal antibodies and thrombotic risk thoroughly. In our study, the use of convalescent hyperimmune plasma was more common among patients who experienced bleeding, in contrast to patients who did not experience bleeding. Coagulation profile of human COVID-19 convalescent plasma was found to be impoverished with coagulation factors and, consequently, has prolonged coagulation time [26]. Such a profile might contribute to hemostasis impairment and higher incidence of bleeding events.

D-dimer levels have been extensively studied in COVID-19. It was recognized as a marker of adverse outcome of infection [27] and as an indicator of VTE. Our analysis showed that high CAR and D-dimer values were predictive of thrombosis occurrence also in the context of CLL. Evidently, coagulopathy in COVID-19 infection, coupled with malignancy related coagulopathy, results in state of highly elevated risk of thrombosis development [28, 29]. A higher D-dimer cut-off level in our cohort of COVID-19 CLL patients corresponded to the higher D-dimer levels found in cancer patients with COVID-19 [30], emphasizing the need for strict follow-up of this specific group of patients. Albumin level, as an acute phase reactant, has been associated with both the adverse outcome of COVID-19 and the development of thrombotic events during COVID-19. Hypoalbuminemia as a consequence of acute or chronic inflammation or increased albuminuria can contribute to the development of thrombosis, because of albumins anticoagulant and antiplatelet characteristics [31–33].

Similar to the general population, the admission to ICU was found to be predictive of thrombosis occurrence. This finding was one of the initial hallmark observations of COVID-19 infection, which has been later extensively confirmed [34–36]. The combination of COVID-19 disease severity of patients in ICU, long list of risk factors related to ICU conditions and treatment and solely patients’ characteristics (including malignancies and comorbidities), lead to a detrimental combination for thrombosis development.

Most CLL patients included in the study were administered thromboprophylaxis (90.5%). In keeping with the literature [37], thromboembolic events were significantly more frequent among CLL patients without thromboprophylaxis than those with thromboprophylaxis. The rate of thromboembolism was lower in patients who were administered prophylactic anticoagulation, in comparison with intermediate and therapeutic anticoagulation. However, this latter finding should be cautiously interpreted considering that higher dosages of anticoagulation therapy were probably administered to the patients with more severe clinical course of COVID-19. In addition, it was shown that the use of thromboprophylaxis is associated with lower mortality rate in severely ill COVID-19 patients [38].

Higher doses of anticoagulation were universally recognized as major drivers of bleeding complications [9, 39, 40]. In our study, 5.0% (23/468) had bleeding events, of which more than 50% were classified as major. Patients treated with intermediate/therapeutic doses of LMWH had a higher rate of bleeding than those treated with prophylactic doses of anticoagulation (8.1% and 3.8%, respectively). That said, the risk of bleeding in these patients depends on numerous factors besides the dosage of anticoagulation therapy, including age, CLL disease status (watch and wait or active disease), severity of COVID-19, comorbidities, and inherited or acquired coagulation abnormalities. Of note, we did not identify any association between CLL-specific treatment with BTK inhibitors and the occurrence of a bleeding episode.

The COVID-19 pandemic has raised questions regarding the changes to therapy for the CLL patients being treated, who tested positive for SARS-CoV2. In our study continuation vs. discontinuation of BTKi was not predictive of thrombosis or bleeding occurrence in the patients with CLL who were receiving BTKi at the time of severe COVID-19 infection. Based on this finding and recently published reports suggesting a possible benefit from the BTKis in the setting of severe COVID-19 infection, and the fact that stopping ibrutinib can result in a disease flare-up in patients with CLL, we may recommend that BTKis therapy should be administered until the risks outweigh the therapy benefits [21, 22].

Our study had several limitations particularly stemming from its retrospective nature, including heterogeneity in the treatment approaches for COVID-19. Additionally, we restricted the analysis to patients with severe COVID-19, thus patients with mild or asymptomatic SARS-CoV-2 infection were not studied.

## Conclusions

In conclusion, patients with CLL diagnosed with COVID-19 are at a high risk of thrombosis if thromboprophylaxis is not used, but also at increased risk of bleeding under the LMWH intermediate/therapeutic dose administration. More collaborative studies are needed to define the optimal anticoagulation treatment strategy that will provide sufficient benefit, without harm, for severely ill CLL patients hospitalized with COVID-19. Age, serum CRP, albumin level, and D-dimer are simple, easily accessible parameters and may be good candidates for defining subgroups of CLL patients who are at increased risk for thrombosis and bleeding during COVID-19.

## Data Availability

The datasets used and analysed during the current study are available from the corresponding author on reasonable request.

## Abbreviations

VTE: Venous thromboembolism
PE: Pulmonary embolism
COVID-19: Coronavirus disease 2019
ICU: Intensive care unit
CLL: Chronic lymphocytic leukemia
ERIC: European Research Initiative on CLL
RT-PCR: Real-time polymerase chain reaction
CIRS: Cumulative illness rating scale
ALC: Absolute lymphocyte count
CRP: C-reactive protein
ECMO: Extracorporeal membrane oxygenation
OR: Odds ratios
CI: Confidence interval
ROC: Receiver operating characteristic
SLL: Small lymphocytic lymphoma
MBL: Monoclonal B-cell lymphocytosis
BTKi: Bruton tyrosine kinase inhibitors
PI3K: Phosphatidylinositol-3-kinase inhibitors
IL-6: Interleukin 6
LMWH: Low-molecular-weight heparin
DOACs: Direct oral anticoagulants
CAR: C-reactive protein to albumin ratio
AUC: Area under curve
ULN: Upper limit of normal
SARS-CoV-2: Severe acute respiratory syndrome coronavirus 2
